# Probio-Ichnos: A Database of Microorganisms with In Vitro Probiotic Properties

**DOI:** 10.3390/microorganisms12101955

**Published:** 2024-09-27

**Authors:** Margaritis Tsifintaris, Despoina Eugenia Kiousi, Panagiotis Repanas, Christina S. Kamarinou, Ioannis Kavakiotis, Alex Galanis

**Affiliations:** 1 Department of Molecular Biology and Genetics, Democritus University of Thrace, 68100 Alexandroupolis, Greece; mtsifintaris@gmail.com (M.T.); dkiousi@mbg.duth.gr (D.E.K.); panrepa@hotmail.com (P.R.); christinakamarinou7@gmail.com (C.S.K.); ikavakiotis@gmail.com (I.K.); 2 Institute of Technology of Agricultural Products, Hellenic Agricultural Organization—DIMITRA, 14123 Lycovrissi, Greece

**Keywords:** probiotics, database, in vitro properties, antimicrobial activity, immunomodulation, antioxidant capacity, antiproliferation, adhesion, strain specificity

## Abstract

Probiotics are live microorganisms that, when consumed in adequate amounts, exert health benefits on the host by regulating intestinal and extraintestinal homeostasis. Common probiotic microorganisms include lactic acid bacteria (LAB), yeasts, and Bacillus species. Here, we present Probio-ichnos, the first manually curated, literature-based database that collects and comprehensively presents information on the microbial strains exhibiting in vitro probiotic characteristics (i.e., resistance to acid and bile, attachment to host epithelia, as well as antimicrobial, immunomodulatory, antiproliferative, and antioxidant activity), derived from human, animal or plant microbiota, fermented dairy or non-dairy food products, and environmental sources. Employing a rigorous methodology, we conducted a systematic search of the PubMed database utilizing the keyword ‘probiotic’ within the abstracts or titles, resulting in a total of 27,715 studies. Upon further manual filtering, 2207 studies presenting in vitro experiments and elucidating strain-specific probiotic attributes were collected and used for data extraction. The Probio-ichnos database consists of 12,993 entries on the in vitro probiotic characteristics of 11,202 distinct strains belonging to 470 species and 143 genera. Data are presented using a binary categorization approach for the presence of probiotic attributes according to the authors’ conclusions. Additionally, information about the availability of the whole-genome sequence (WGS) of strains is included in the database. Overall, the Probio-ichnos database aims to streamline the navigation of the available literature to facilitate targeted validation and comparative investigation of the probiotic properties of the microbial strains.

## 1. Introduction

Probiotics are defined as live microorganisms that may confer health benefits on the host when administered in adequate amounts [1]. This definition is supported by experimental and clinical data that showcase the capacity of specific probiotic strains to preserve and restore intestinal and extraintestinal homeostasis [2,3]. Specifically, positive outcomes have been demonstrated for the management of intestinal and systemic diseases, including lactose intolerance [4], Crohn’s disease and ulcerative colitis [5], antibiotic-associated diarrhea [6], osteoporosis and other bone-related diseases [7], as well as neurodegenerative [8], autoimmune [9], mood and behavioral disorders [10,11]. Additionally, probiotics have also been investigated as preventive or adjunctive therapeutic agents for cancer [12,13]. Probiotics may provide these beneficial effects by exerting antimicrobial [14,15], antiproliferative [16], or antioxidant potential [17] or by modulating immune system responses [18].

Probiotic microorganisms, mainly lactobacilli, bifidobacteria, and Bacillus species, can be isolated from fermented dairy or non-dairy products [19], host niches, including human saliva, feces [20], and breast milk [21] or environmental sources, such as soil [22] or plant epiphytic communities [23]. Their characterization and classification into taxonomic groups is performed with amplicon sequencing targeting housekeeping genes, commonly the 16S rDNA [24]. The integration of the WGS platforms in probiotic research has enhanced the accuracy of these phylogenomic studies and has subsequently led to the recent reclassification of several LAB [25]. Preliminary in vitro testing is performed according to specific guidelines, established initially by FAO/WHO [1] and later revised by the International Scientific Association for Probiotics and Prebiotics (ISAPP) [26], to evaluate the safety profile and probiotic traits of the novel isolates. In addition, complete-genome-sequence analysis is required by EFSA for strains intended to enter the food chain [27].

**Figure 1:**
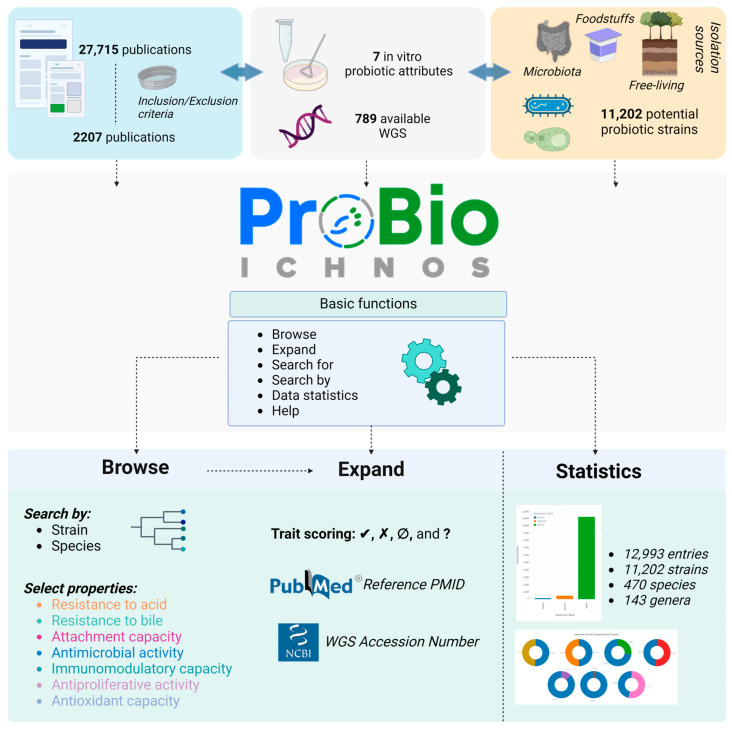
Overview of the Probio-ichnos database. The Probio-ichnos database is constructed using the available literature on in vitro probiotic properties of strains isolated from human, animal, or plant microbiota, as well as food products and the environment. This data is derived from a total of 2207 publications on 11,202 bacterial and fungal strains examined for basic probiotic attributes: resistance to acid and bile, attachment to host epithelia, as well as antimicrobial, immunomodulatory, antiproliferative, and antioxidant activities. Users can browse the database and search by species or the strain name for all the probiotic traits or for selected attributes. The traits for each strain are scored using a qualitative method based on the authors’ conclusions. The ‘Browse’ and ‘Expand’ functions support the identification of the available literature and genomic information by redirecting the user to the PubMed and the NCBI Assembly databases. All the datasets can be directly downloaded in a JSON format from the ‘Help’ page.

Although the number of studies on the health-related effects of probiotics has increased significantly, further research is needed to explore their strain-, host- and disease-specific activities and define their molecular mechanisms of action [28]. The application of bioinformatic tools, the development of new methodologies and pipelines, as well as the construction of specialized databases of probiotic microorganisms can contribute significantly to this endeavor. To this end, we have developed the Probio-ichnos database (https://probio-ichnos.streamlit.app/) (accessed on 2 September 2024), where “ichnos” is the Greek word for “fingerprint”. This is a manually curated, literature-based database that aims to support probiotic research by collecting and comprehensively presenting in vitro data for potential probiotic strains isolated from diverse niches. Specifically, our database is focused on seven probiotic characteristics: the ability to survive in the gastrointestinal tract (i.e., resistance to acid and bile salts), attachment to and transient colonization of the gut, as well as antimicrobial, immunomodulatory, antiproliferative, and antioxidant activity (Figure 1). Additionally, the database catalogs strains with draft or complete genome sequences deposited at public repositories. The Probio-ichnos database has a user-friendly interface designed for ease of navigation and access to relevant data, allowing the interactive exploration of database entries. Users can discover strains with replicable findings, identify gaps or inconsistencies in the available experimental data, explore novel methodologies and protocols, and perform comparative studies between the strains of interest. Ultimately, the Probio-ichnos database represents a useful tool for researchers in the field, as it facilitates the comprehensive exploration of the in vitro probiotic properties of microorganisms.

## 2. Materials and Methods

### 2.1. Data Collection and Processing

To collect information on microorganisms with probiotic characteristics, the PubMed database was searched using the advanced search builder and the query “(probiotic[Title/Abstract])”. Next, a list of bacterial and fungal species was obtained from the NCBI Taxonomy Database and was utilized to filter the records to include all the known microorganisms. Records of clinical studies or human trials, reviews (systematic or narrative), and metanalyses were excluded.

### 2.2. Abstract Screening and Data Extraction

A manual abstract screening process was employed to build the Probio-ichnos database. The article abstracts were screened and selected according to three criteria: (i) taxonomic classification at the strain level, (ii) the investigation of in vitro probiotic attributes, and (iii) the use of single strains and not of probiotic combinations or synbiotics. The main body and the Supplementary Materials of the selected articles were manually reviewed to extract information on the in vitro probiotic attributes of microorganisms, including their capacity to withstand the conditions of the gastrointestinal tract (stomach acid and bile salts), their adherence/attachment capacity, as well as their antimicrobial (activity against bacteria, fungi, and viruses), immunomodulatory (either pro- or anti-inflammatory), antiproliferative (or cytotoxic), and antioxidant activity. A qualitative categorization approach was employed to document the presence of a probiotic attribute in the included literature. More specifically, the authors’ conclusions for each strain were recorded as follows: Yes—if the strain presented the feature; No—if the strain did not present the feature; None—if the strain was not tested for the specific feature. In cases where multiple entries were present for a microorganism, a scoring system was implemented. A score of +1 was allocated for an affirmative result (‘Yes’), while a score of −1 denoted a negative result (‘No’). This scoring methodology facilitated a comprehensive evaluation of the cumulative evidence. Subsequently, the collective score underwent meticulous scrutiny; if the aggregate score exceeded zero, the strain was confidently classified as exhibiting the feature (‘Yes’). Conversely, if the score dipped below zero, it was determined that the strain did not possess the attribute (‘No’). A score of zero was indicative of inconclusive evidence regarding the presence or absence of the feature, leading to the classification as ‘Ambiguous’. Finally, the NCBI Taxonomy database was utilized to identify the current nomenclature, and the NCBI Assembly database was used to extract the available WGSs of strains included in the database. Specifically, for lactobacilli, the updated taxonomy and nomenclature proposed by Zheng et al. (2020) was followed [25].

### 2.3. Implementation

The Probio-ichnos database was constructed using the Streamlit framework (https://streamlit.io/) (accessed on 6 May 2024), an open-source app framework in Python language. For data visualization, we leveraged Altair (https://altair-viz.github.io/) (accessed on 6 May 2024), a declarative statistical visualization library in Python. Throughout the development process, rigorous testing was conducted on major browsers such as Microsoft Edge, Google Chrome, and Mozilla Firefox to ensure cross-compatibility and an optimal user experience across the platforms.

## 3. Results

### 3.1. Data Collection, Processing and Extraction

**Figure 2:**
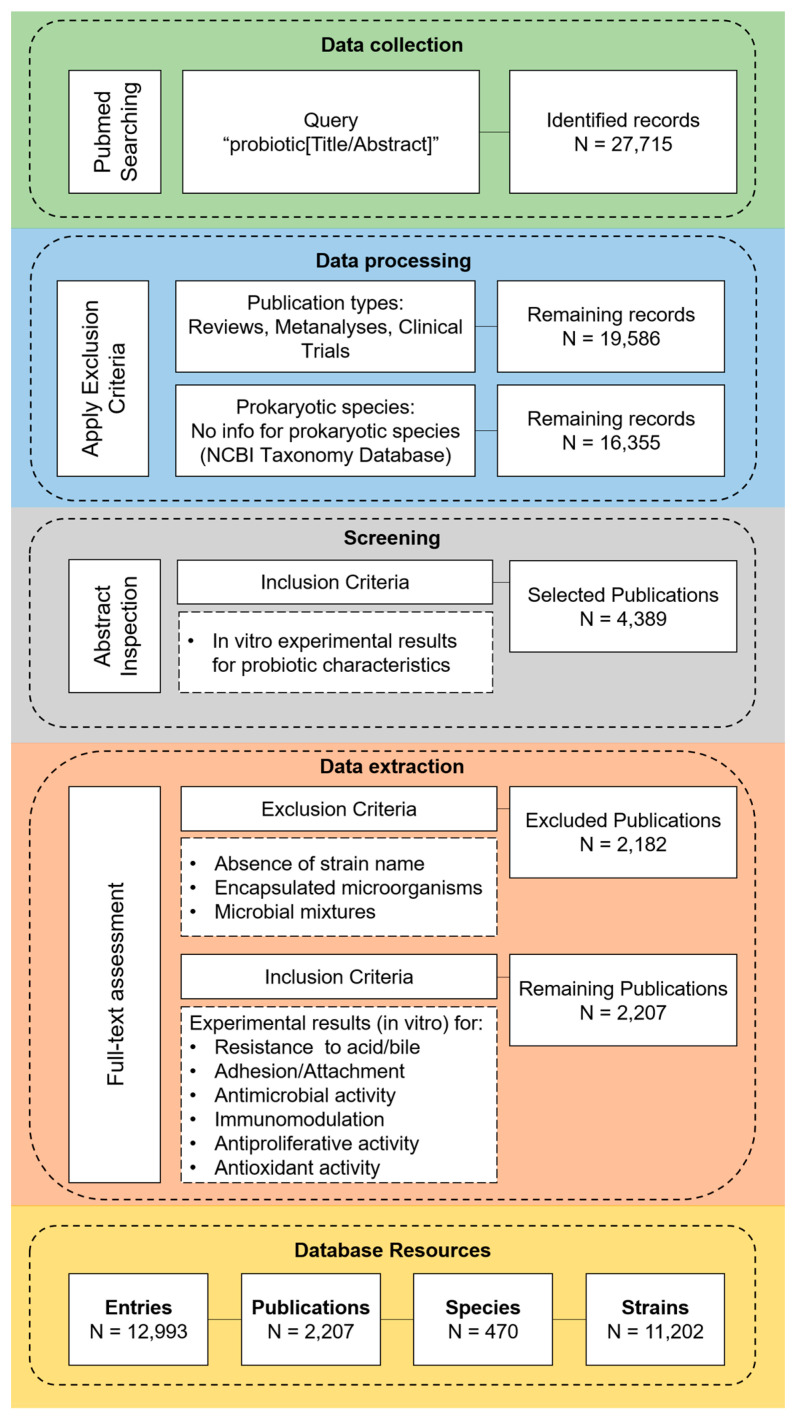
Schematic representation of the data extraction methodology followed for the construction of the Probio-ichnos database.

The initial search with the query “(probiotic[Title/Abstract])” generated a total of 27,715 records, encompassing data until January 2024. Narrative, systematic reviews and clinical studies were excluded, resulting in 19,586 remaining records. Next, the hits were filtered using the NCBI Taxonomy list, and records with no information about the archived species were excluded (n = 3231). From the remaining 16,355 publications, only 4389 contained data derived from in vitro studies on the probiotic potential of isolates and were included in the data extraction phase. Subsequently, the full text and the Supplementary Materials of the records were meticulously reviewed based on strict inclusion/exclusion criteria (Figure 2). The final database comprised 12,993 unique entries sourced from a total of 2207 publications, providing information on 11,202 strains. The flowchart, outlining the methodology and detailing each step of the screening, filtering, and compiling of the final database resources, is shown in Figure 2.

### 3.2. Database Interface and Modules

**Figure 3:**
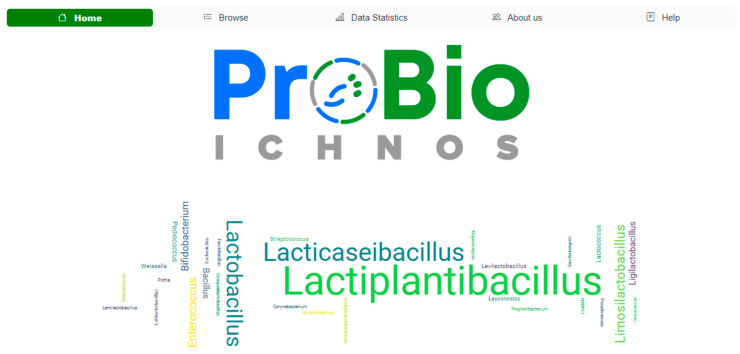
The ‘Home Page’ of the Probio-ichnos database contains an interactive word cloud depicting the distribution of entries across the different genera. The size of each word is indicative of the number of entries associated with the specific genera.

The Probio-ichnos database provides a number of functionalities to enhance user experience and facilitate the evaluation of the probiotic attributes of the novel isolates and of the commercially available strains. The Probio-ichnos database is freely accessible via a web browser at https://probio-ichnos.streamlit.app/ (accessed on 2 September 2024). The ‘Home Page’ serves as a welcoming gateway to the database, offering the users an engaging visual representation of its contents. At the forefront is an interactive word cloud, a dynamic display that visually depicts the distribution of the database entries across the different genera (Figure 3), where the size of each word corresponds to the number of entries associated with that particular genus. Hovering over a genus in the word cloud, the users are provided with details such as the total number of entries, species, and strains associated with that specific genus, enhancing the interactive nature of the visualization and enabling deeper exploration of the database.

**Figure 4:**
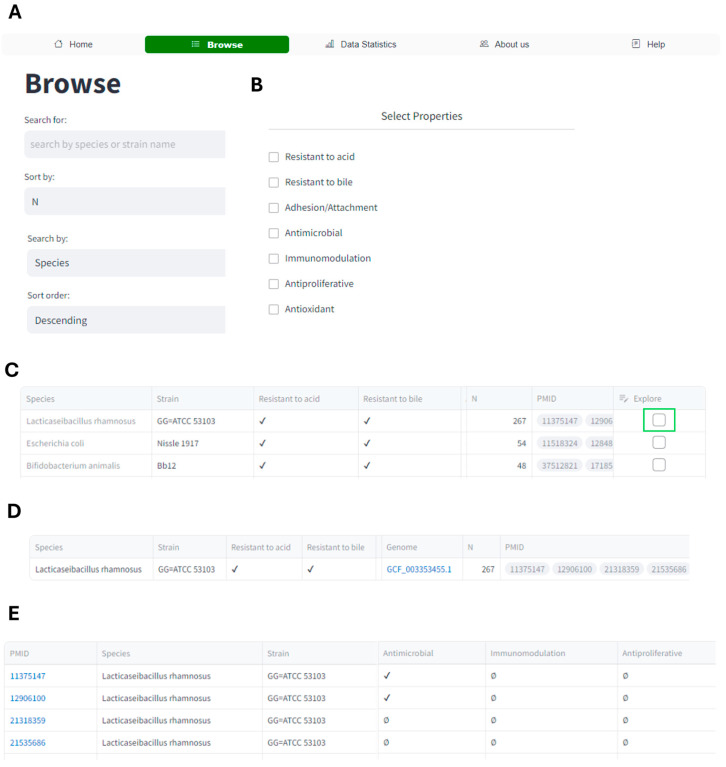
Entries can be explored in the ‘Browse’ tab of the Probio-ichnos database. (**A**) The database can be searched using the species or the strain name and/or (**B**) by selecting specific probiotic attributes. (**C**) The main table consists of rows that contain a strain and the probiotic properties, the number of studies available, and their PMID. Detailed exploration of a specific strain and its attributes can be achieved by ticking the ‘Explore’ option in the ‘Browse’ tab, highlighted in green. By selecting the ‘Expand’ option, two new tables are generated: (**D**) a table containing the main results for the specific strain and (**E**) a table of entries of the individual records. By selecting the unique PMID, the user is redirected to the article from which the data were extracted. Accordingly, the availability of the WGS of strains is indicated in the top table; by clicking on the link, the user is redirected to the NCBI Assembly database.

The ‘Browse’ page of the Probio-ichnos database offers users a comprehensive platform with which to explore the microbial strains and their probiotic properties (Figure 4). Upon entering the species or the strain name into the search box (Figure 4A), the users receive the results in the form of a table displaying the microorganisms along with their respective properties. This search capability allows users to query the database by species or strain name, providing concise information solely on the presence or absence of the specific properties associated with the microorganisms stored within the database. Beneath the search bar, the checkboxes corresponding to each probiotic property enable users to filter the table, displaying the microorganisms with the selected properties (Figure 4B). Users have the flexibility to choose multiple properties simultaneously, tailoring their search to specific criteria. Each row in the table (Figure 4C) represents a microorganism along with its properties, with the “PMID” column containing the PubMed database IDs of the studies referenced for each entry. The “N” column indicates the number of studies cited for each entry. The symbols ✔, ✗, ∅, and ? are employed within the database to indicate whether an in vitro property has been recorded in the literature for a specific strain: the symbol ‘✔’ indicates that the strain exhibits the property, while ‘✗’ indicates that the strain does not exhibit the property, based on the authors’ conclusions. In cases where there are no records available for the property, ‘∅’ is used, and ‘?’ is employed to denote uncertainty regarding whether the strain exhibits a specific phenotype. These symbols provide users with clear and concise information regarding the probiotic properties of each strain listed in the database, facilitating the efficient interpretation of the data. Moreover, navigation buttons enable users to conveniently navigate through the entries, allowing them to view the next set of 10 entries in the table with each click. The interface also provides information on the total number of entries resulting from the applied filters. Additionally, users can delve deeper into the database by accessing all the publications associated with a particular microorganism. This is facilitated by selecting a checkbox in the ‘Explore’ column of the table and clicking the ‘Expand tab’. Figure 4D shows the results obtained when exploring publications associated with the microorganism *Lacticaseibacillus rhamnosus* GG (LGG). The first table represents the selected row in the main table, while the second table displays detailed information on all the studies related to the chosen microorganism. By clicking the PubMed database ID in the first column, users can directly access the publication used to extract the data. Additionally, a link to the NCBI Assembly database for the strains with available WGS is provided.

Comprehensive documentation is displayed on the ‘Help’ page of the Probio-ichnos database. Furthermore, users can download the database data in a JSON format directly from this page. This feature enhances accessibility and enables users to utilize the data for further analysis or integration into their own research projects.

### 3.3. Data Statistics

**Figure 5:**
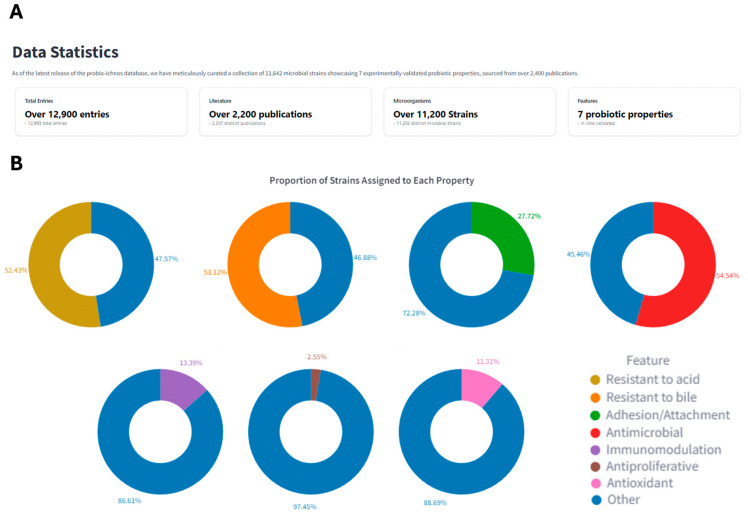
(**A**) Data statistics. (**B**) The distribution of the strains into the seven probiotic categories: resistance to acid, resistance to bile, adhesion/attachment, as well as antimicrobial, immunomodulatory, antiproliferative, and antioxidant capacities. Strains that did not exhibit or were not studied for a specific trait are clustered in ‘Other’.

The Probio-ichnos database boasts a large collection of microbial strains, namely, 11,202 individual strains belonging to 470 distinct species and 143 different genera. The top 20 entries at each taxonomic rank within the Probio-ichnos database are *Lactiplantibacillus* (n = 2086) for the genus level, *L. plantarum* (n = 1839) for the species level, and LGG (n = 267) for the strain level (Figure S1). Seven key probiotic attributes are presented in the database: resistance to acid (5873 strains), resistance to bile (5950 strains), adhesion/attachment capability (3105 strains), antimicrobial capacity (6110 strains), immunomodulatory activity (1500 strains), antioxidant activity (1267 strains), and antiproliferative activity (286 strains). Resistance to acid and bile are the two most common attributes presented in the studies, followed by antimicrobial capacity (Figure 5).

### 3.4. Case Study

To demonstrate the capabilities of the Probio-ichnos database, we conducted a case study for the strain *Lactobacillus acidophilus* LA-14. To this end, we queried the database for entries with the code ‘La-14’ under the ‘Strain’ category. This search returned nine entries derived from the research papers that fulfill the criteria demonstrated in Figure 2. By selecting the strain of interest and the ‘Expand’ tab, a comprehensive list of experimental findings and links to relevant literature and to the NCBI Assembly database were provided. As shown in Figure S2, the strain has been studied for five in vitro probiotic characteristics. Specifically, the available studies show that the strain can withstand acid and bile, attach to epithelial cells, and exert antimicrobial and immunomodulatory activity. The antioxidant and the antiproliferative potential of the strain has not been reported in the literature yet, as indicated by the ‘∅’ symbol (Figure S2A). By following the PMID links (Figure S2B), users can assess the study design, protocols, and experimental results that support the authors’ conclusions for each trait. Additionally, a link to the WGS of the strain is provided (GCF_000389675.2). Subsequently, we wanted to compare the results produced by the Probio-ichnos database with those generated by the PubMed database. Thirty entries were generated with this search, among which only three were shared with the Probio-ichnos database (PMIDs: 22143809, 37404182, 37621875). Indeed, most of the articles did not contain the relevant information on the in vitro probiotic potential of the strain *Lactobacillus acidophilus* La-14. Specifically, thirteen articles test synbiotics or a mixture of different strains and/or exclusively contain in vivo or clinical data (PMIDs: 37629672, 34540177, 34111674, 33765904, 31941108, 31250099, 29974728, 28545241, 26749248, 25855055, 22459313, 18422632, 36838464), six investigated microencapsulation efficiency or did not test for probiotic traits (PMIDs: 32849418, 30210464, 30150831, 25837504, 17499563, 34600952), one was an article correction (PMID: 28686189) and two were genome announcements (PMID: 25593259, 23788546). For one, the full text was not available at the time of the database construction (PMID: 24913839), two did not include the strain *Lactobacillus acidophilus* La-14 in the main text or in the Supplementary Materials (PMID: 24126832, 34592620), and one was not available in English (PMID: 24020254). Apparently, the Probio-ichnos database can significantly streamline the navigation of the available data and, therefore, increase the efficiency of literature searches. Importantly, six articles were only identified by the Probio-ichnos database and not by the PubMed database. This is attributed to two factors: the strain, *Lactobacillus acidophilus* La-14, was not included in the title, abstract or keywords (PMIDs: 36688748, 23102956, 36838294), or the strain code was written with spelling variations (e.g., La14) (PMIDs: 32403331, 25729438, 25366201). Therefore, the manual screening and extraction of data rendered the Probio-ichnos database a comprehensive resource for literature on probiotic microorganisms with in vitro probiotic potential.

## 4. Discussion

Probio-ichnos is the first comprehensive database containing literature-derived, manually curated information on selected in vitro probiotic properties of microbial strains. Specifically, the capacity to withstand simulated GI tract conditions, attachment to host epithelia, as well as antimicrobial, immunomodulatory, antioxidant, and antiproliferative activities are included, as these are some of the most well-studied in vitro characteristics involved in probiotic action [28]. For the construction of the database, we searched PubMed, a freely accessible database that contains more than 37 million citations and covering a large number of reputable journals in the field, for entries with the keyword ‘probiotic’ in the title and/or abstract. Of note, the PubMed database has been exclusively used for literature extraction in other probiotic-related databases, such as the FermFooDb [29], and the ProbResist [30]. The search returned more than 27,000 results. Using a series of inclusion and exclusion criteria, relevant papers were selected and used for data extraction. Currently, more than 12,900 entries from 2207 papers for 11,202 strains are included in the database. The use of the Probio-ichnos database does not require bioinformatic skills, as the site is designed to be user-friendly and interactive. Users can select to view individual traits for all or specific strains; they can also perform combined searches. For example, they can select to view all the lactobacilli with the capacity to withstand bile salts and present antimicrobial activity. Therefore, the Probio-ichnos database supports the comparative analysis of traits and/or strains of interest.

As probiotic action is strain-specific, studies with no information at the strain level were excluded from the Probio-ichnos database. Notably, LGG is the strain with the most entries (n = 267), presenting all probiotic attributes. Indeed, it is one of the most well-studied probiotics at the preclinical and clinical levels and is already included in numerous studies as a reference strain [31]. In this context, the database can be used to identify other strains with replicable findings that can be incorporated into experimental design as control or reference strains.

During the manual construction of the Probio-ichnos database, we observed that the protocols and experimental procedures varied significantly between the studies investigating the same phenotype. At the same time, no official cut-offs or experimental values exist to evaluate the capacity of the strains to attach to the gut epithelium or to exert antimicrobial, immunomodulatory, antioxidant, or antiproliferative effects. For this reason, we selected to present the available data based on the authors’ conclusions and developed a scoring method to determine the replicability of the findings. The symbols “✔” and “✗” are employed within the database to indicate the presence or absence of a feature, while the symbol “∅” indicates that the specific phenotype has not been previously reported in the literature. Therefore, our database supports the identification of gaps in our knowledge about probiotic attributes of the strains of interest, thus supporting discovery in the field. For strains with ambiguous experimental findings, the symbol “?” is employed. This suggests possible discrepancies between the studies, underlining the importance of controlled and standardized experimental procedures. It is important to note that most of the strains included in the Probio-ichnos database are only investigated in a single article (10,447 out of 11,202 strains), highlighting the need for independent replication studies.

The Probio-ichnos database also provides information on the availability of the WGSs of the strains in a separate table. Notably, out of a total of over 11,000 strains included in our database, only 789 have their WGSs deposited in the NCBI Assembly database. The WGS is fundamental for the correct taxonomic classification of novel isolates into species and to ensuring that they are not already deposited under a different strain name in public repositories. Indeed, the accumulation of bacterial WGSs has led to the recent reclassification of the former *Lactobacillus* genus into 25 genera with shared ecological and metabolic properties [25]. Furthermore, the whole-genome analysis contributes to the elucidation of the safety profile of the strains intended for human use, as the presence of virulence and antibiotic-resistant genes can be determined in silico. Transferable antibiotic-resistant elements have already been identified in the LAB traditionally used in fermented products, highlighting the need to reassess their safety profile using molecular approaches [32]. For this reason, as of 2021, the EFSA requires the WGS of the strains intentionally added to the food chain [27]. Moreover, as the number of available WGSs is continuously increasing, it has become apparent that 50–60% of the annotated proteins in the LAB are hypothetical with no attributed functions [33]. The functional annotation of these proteins could facilitate a better understanding of the biological properties and interactions of probiotics with other microorganisms and the host. To this end, advances in genomic research of microorganisms have supported the correlation between the genomic features and the probiotic phenotype. Tools harnessing artificial intelligence (AI) and machine learning (ML) algorithms have been developed to accurately predict the probiotic potential of novel isolates based on their WGSs. In this context, the iProbiotics [34] and the ProbioMinServer [35] platforms have been developed using ML and existing algorithms (i.e., RAST, KEGG, CAZy) to study probiotic-related attributes, including host adaptation, metabolic capacity, drug resistance, and virulence activity in silico. In this context, the Probio-ichnos database is a comprehensive resource for WGS of potential probiotic strains that can be used as inputs for these tools.

The Probio-ichnos database complements existing databases and search engines by covering a wide spectrum of phenotypes related to the in vitro probiotic phenotype. The MASI database includes information about the reciprocal relationship between probiotics and the gut microbiota [36], while ODRAP includes information about the probiotic–diet interactions and comprehensively presents the capacity of prebiotics to induce the proliferation of specific bacterial species, including probiotic microorganisms [37]. In addition, the FermFooDd database presents bioactive compounds in foodstuffs that are derived from the metabolic activity of probiotics and starter cultures in the food matrix [29]. Other databases are dedicated to specific probiotic attributes: OCINS is primarily concerned with antimicrobial peptides produced by lactobacilli, bifidobacterium, and enterococci [38], and the ProbResist database catalogs probiotic strains with experimentally proven resistance to antibiotics [30]. Additionally, ProBioQuest, a search engine that includes literature, patent filings, and clinical data on probiotics derived from open-access sources, has been developed to aid the study of the health-promoting properties of probiotics [39]. The simultaneous and combinatorial use of the available tools and repositories can support the holistic study of the biology, interactions, and strain-specific health-promoting effects of probiotics on the host.

## 5. Conclusions

The Probio-ichnos database is the first manually curated, literature-based database that collects and comprehensively presents in vitro probiotic attributes of bacteria and fungi. The database consists of 12,993 entries for seven probiotic characteristics (i.e., tolerance to acid and bile salts, adherence capacity, as well as antimicrobial, antioxidant, immunomodulatory, and antiproliferative activity) of 11,202 strains belonging to 470 species and 143 genera. The database is user-friendly and easy to navigate, thus facilitating access to the relevant literature. The Probio-ichnos database aims to advance the understanding of strain-specific probiotic action by supporting targeted validation and comparative investigation of the probiotic properties of microbes derived from diverse niches.

## Data Availability

The Probio-ichnos database and its content are freely accessible to all users at https://probio-ichnos.streamlit.app/ (accessed on 2 September 2024). Users can download the datasets through the ‘Help page’.

## Supplementary Materials

The following supporting information can be downloaded at https://www.mdpi.com/article/10.3390/microorganisms12101955/s1, Figure S1: Top 20 entries at the (a) genus, (b) species, and (c) strain level within the Probio-ichnos database. Figure S2: Results derived from the Probio-ichnos database for strain *Lactobacillus acidophilus* LA-14. (a) *Lactobacillus acidophilus* LA-14 is included in 9 articles, and it presents resistance to acid and bile, adherence capacity, and antimicrobial and immunomodulatory activities based on the authors’ conclusions. (b) By clicking the ‘Expand’ tab, the user is redirected to new tables containing individual entries for each of the nine papers, as well as links to the PubMed and the NCBI Assembly databases.
