# Supplementary figures and images for: Probio-Ichnos: A Database of Microorganisms with In Vitro Probiotic Properties
Source: Microorganisms. 2024 Sep 27;12(10):1955. doi: 10.3390/microorganisms12101955 (PMC11509836; doi:10.3390/microorganisms12101955)

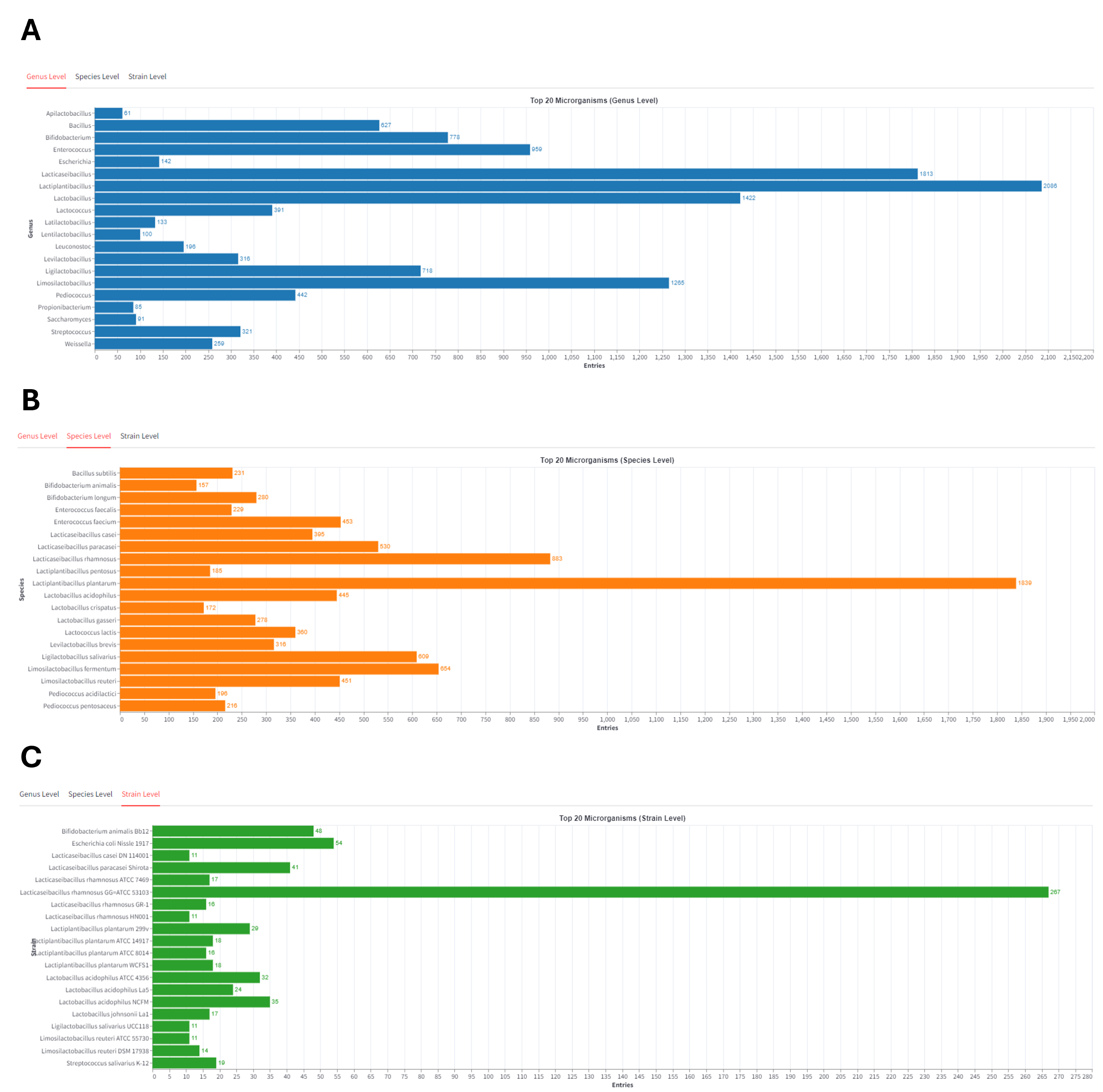

Supplement: Supplementary file 1 [file microorganisms-12-01955-s001.zip › Figure S1.png]

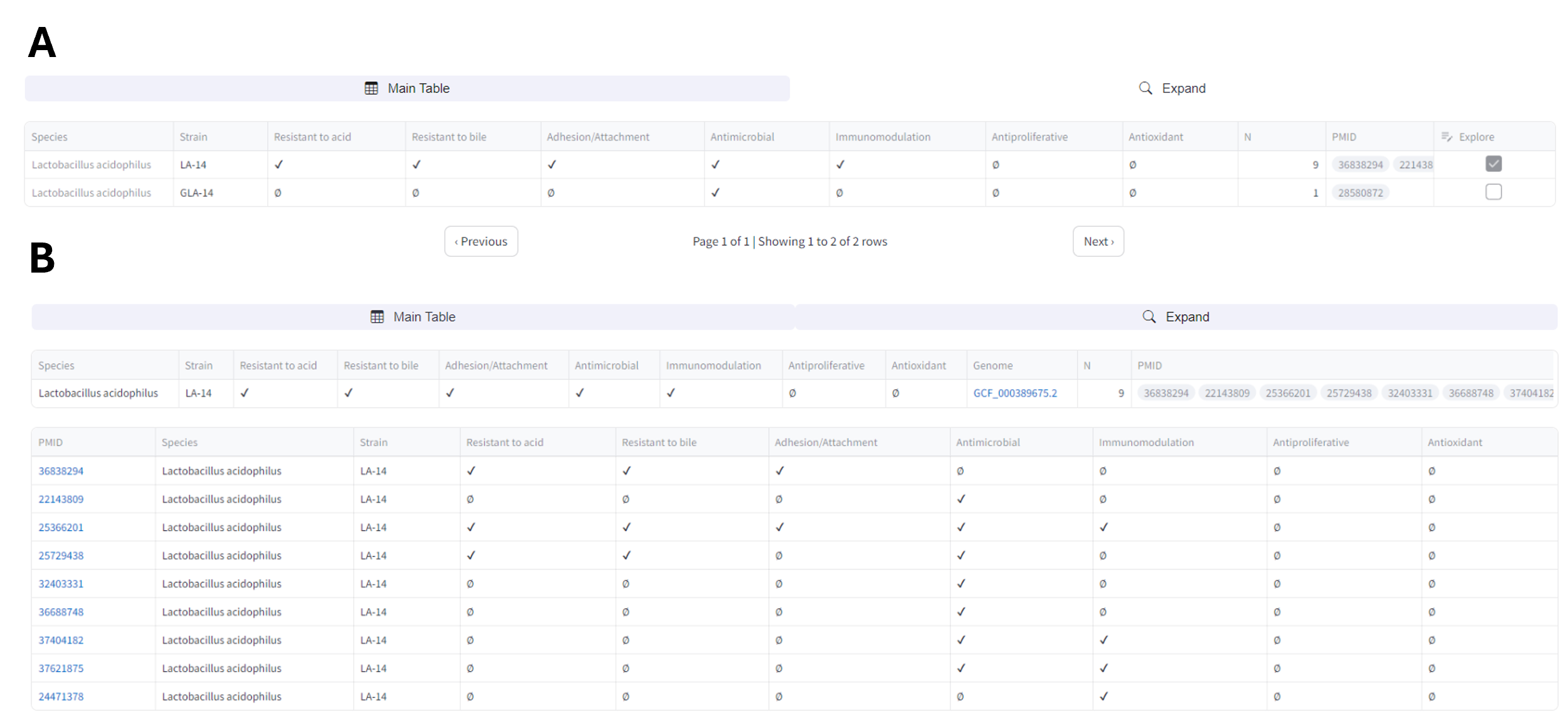

Supplement: Supplementary file 1 [file microorganisms-12-01955-s001.zip › Figure S2.png]
